# Seize Substitute Specifics

**Published:** 1915-12

**Authors:** 


### Seize Substitute Specifics.

CHEAP IMITATIONS OF WELL-KNOWN PREPARATIONS PEDDLED TO DRUG STORE PROPRIETORS.

WASHINGTON, D. C.

Several shipments of worthless imitation drug products have been seized by the officials in charge of the enforcement of the Food and Drugs Act. Itinerant peddlers are selling to drug stores large quantities of preparations made up and labeled in imitation of high-priced patent medicines of foreign origin. Only small quantities of the genuine medicines have been imported since the war began, causing a great increase in prices. Unscrupulous manufacturers are attempting to reap a harvest by substituting for the genuine medicines cheap chemicals with no medicinal value whatever. In order to make it difficult to trace these preparations to the parties responsible for their manufacture, they are not usually distributed through the regular channels of commerce, but are peddled about to drug stores by itinerants who make immediate delivery at the time of sale.

A preparation put up in imitation of "Neosalvarsan," a medicine which has largely displaced the preparation known as 606 in the treatment of syphilis, is being distributed to drug stores in this manner. A sample labeled "Neosalvarsan," which was recently examined by the Department, was found to be nothing more than salt colored with a coal-tar dye, none of the genuine neosalvarsan whatever being present. The label on this product was an exact reproduction of the genuine imported salvarsan, or it was an original container refilled with the imitation articles.

This fraud is held to be particularly flagrant, according to the medical experts of the Department, not alone because a worthless preparation is sold for a high price, but mainly because neosalvarsan is usually administered by injection directly into the blood of the syphilitic patient. The cheap substitute is not only worthless in the treatment of this disease, but when injected directly into the blood might work considerable injury.

Other preparations which are peddled to druggists and purport to be acetylsalicylic acid, commonly known as aspirin, a medicine of foreign origin regularly prescribed by many physicians for certain ailments, have been seized by the officials in charge of the enforcement of the Food and Drugs Act, because an analysis showed that the products were worthless imitations.

Owing to the manner in which these preparations are peddled about, it is difficult to trace the interstate shipment of any of them, and in cases where there has been no interstate shipment the Federal Food and Drugs Act has no jurisdiction. On information furnished by the Federal authorities some of these imitation goods have been seized by city officials who had authority under State laws to proceed when there had been no interstate shipment.

